# CoQ_10_ enhances PGC1α and increases expression of mitochondrial antioxidant proteins in chronically ischemic swine myocardium

**DOI:** 10.1186/s12986-019-0418-8

**Published:** 2019-12-30

**Authors:** Laura Hocum Stone, Erin Chappuis, Christin Wright, Rosemary F. Kelly, Edward O. McFalls

**Affiliations:** 1 Department of Surgery, University of Minnesota Twin Cities, Minneapolis, USA; 2 Research Service, Minneapolis VA Medical Center, Minneapolis, USA; 3 Department of Medicine, University of Minnesota Twin Cities, Minneapolis, USA; 4 Cardiology (111C), Minneapolis VA Medical Center, 1 Veterans Drive, Minneapolis, MN 55417 USA

**Keywords:** Hibernating myocardium, CoQ_10_, Mitochondria, PGC1α

## Abstract

**Background:**

Expression of mitochondrial proteins is reduced within hibernating myocardium (HM). It is unclear whether dietary supplementation with CoQ_10_ can increase expression of mitochondrial electron transport chain (ETC) and antioxidant proteins within this tissue. In a swine model of HM, we tested whether dietary administration of CoQ_10_ for four weeks enhances the expression of ETC and antioxidant proteins within the mitochondria via increased PGC1α signaling.

**Methods:**

12 swine were instrumented with a fixed constrictor around the LAD artery to induce gradual stenosis. At three months, transthoracic ECHO was performed to confirm the presence of a wall motion abnormality in the anterior wall. Animals were then randomly assigned to receive daily dietary supplements of either CoQ_10_ (10 mg/kg/day) or placebo for four weeks. At this time, animals underwent a final ECHO and terminal procedure. Expression of nuclear-bound PGC1α (Western blots) and mitochondrial proteins (Tandem Mass Tag) were determined.

**Results:**

Mitochondrial and nuclear membranes were isolated from the LAD region. Nuclear-bound PGC1α levels were > 200-fold higher with administration of four weeks of CoQ_10_ treatment (*p* = 0.016). Expression of ETC proteins was increased in those animals that received CoQ_10_. Compared with mitochondria in the LAD region from placebo-treated pigs, CoQ_10_-treated pigs had higher levels of Complex I (*p* = 0.03), Complex IV (*p* = 0.04) and Complex V (*p* = 0.028) peptides.

**Conclusions:**

Four weeks of dietary CoQ_10_ in HM pigs enhances active, nuclear-bound PGC1α and increases the expression of ETC proteins within mitochondria of HM tissue.

## Introduction

Coronary artery disease (CAD) is a leading cause of death in the United States. While the mortality rate associated with CAD has gone down in recent years, its incidence and effect on patient quality of life remains high [1]. A subset of CAD patients present with chronically ischemic myocardium that remains viable despite reduced blood flow and regional function at rest. This is known as hibernating myocardium (HM), and is an attractive target for novel therapies due to the presence of viable tissue despite chronic ischemia. Without treatment, HM can eventually progress to heart failure as cardiac function becomes increasingly depressed, especially under chronic ischemic events or during increased workload [2, 3]. The current optimal therapy for HM is timely, complete revascularization to restore blood flow and avoid heart failure. The procedure that best provides complete revascularization is coronary artery bypass surgery (CABG). If revascularized, HM has the potential for myocardial recovery and improved survival. However, although revascularization of HM should conceptually restore contractile function to normal, clinical observations and studies from our lab demonstrate that recovery is often incomplete [4–9].

We have developed and characterized a pig model of HM that recreates the clinical experience of HM as defined by Rahimtoola [10], including reduced blood flow, reduced regional function, and preserved viability as measured by increased glucose uptake [8, 9, 11–14]. Using our animal model, we have identified hallmark adaptations in HM tissue which center around dysregulation of mitochondrial morphology, proteome, and function. Specifically, we have shown that complexes of the electron transport chain (ETC) and PGC1α, a driver of mitochondrial biogenesis, are downregulated in HM and not restored by the standard therapy of revascularization with CABG [15]. As the heart is critically dependent on mitochondrial health to create ATP and meet the energetic demands of the myocytes, the persistent impairment of the mitochondrial proteome must be addressed. This suggests that to enable complete functional recovery within HM regions, enhanced mitochondrial biogenesis, a process involving fission, fusion and autophagy, may be needed [16–20].

PGC1α is also reduced within aging muscle, leading to increased oxidant stress within the tissue [21]. Interestingly, PGC1α levels can be increased nearly three-fold by administration of coenzyme Q_10_ (CoQ_10_) or ubiquinone, as shown in a rat model of neurodegenerative disease, with an observed reduction in oxidant stress markers [22]. CoQ_10_ is a component of Complex III and the Q-cycle of the mitochondrial ETC, and is essential for ATP production, while reducing the accumulation of reactive oxygen species (ROS) [23]. In a swine model, dietary supplementation of CoQ_10_ (10 mg/kg/day) for 30 days increased the myocardial content of CoQ_10_ in isolated mitochondria by 30%, preserved regional function following regional ischemia-reperfusion, and reduced levels of malonaldehyde (MDA) content, a marker of oxidant stress within the tissue [24].

In light of the fact that mitochondrial and functional impairment persists following the standard treatment of CABG, there is a clinical need for new therapies that target the mitochondrial basis of HM. Considering the importance of mitochondrial biogenesis within HM, the purpose of the present study is to determine whether chronic dietary administration of CoQ_10_ would increase the expression of ETC proteins within HM, potentially by a mechanism involving PGC1α, thus addressing the mitochondrial dysfunction that is persistent in HM despite successful CABG.

## Methods

### Study design

**Fig. 1:**
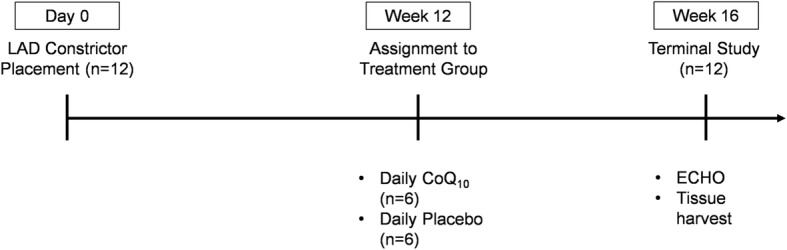
Experimental Design

Twelve animals were subjected to initial instrumentation with a rigid constrictor on the LAD to induce the HM phenotype over a period of twelve weeks. At 12 weeks, the animals were randomly assigned to two treatment groups – CoQ10 or Placebo. Each group received dietary administration of either CoQ10 or a placebo daily for 30 days. At the end of the treatment period, cardiac function was measured by ECHO and the animals were sacrificed for proteomic studies (Fig. 1).

### Swine model of chronic myocardial ischemia

Twelve Yorkshire-Landrace swine were sedated with telazol (4 mg/kg; IM) and xylazine (2 mg/kg; IM), intubated and anesthetized with isoflurane (2%). Using a left thoracotomy approach, a plastic c-shaped constrictor with an internal diameter of 1.5 mm was placed on the LAD artery proximal to the first diagonal without occluding the vessel and secured with sutures. Growth of the animal over the next 12 weeks creates a gradual stenosis of the LAD. Twelve weeks following instrumentation, a transthoracic ECHO was performed to confirm the presence of an anterior wall motion abnormality in the LAD distribution. With confirmation of chronically ischemic heart tissue, pigs were randomly assigned to receive daily dietary supplements of either CoQ_10_ (10 mg/kg/day) (*n* = 6) or placebo (n = 6) for four weeks. At 16 weeks, animals underwent a terminal procedure.

### Terminal procedure

Animals were sedated, anesthetized and ventilated, as outlined in the initial procedure. A transthoracic ECHO was performed to assess regional wall thickening under baseline rest conditions and dobutamine infusion (10 μg/kg/min). Cut downs of the carotid artery were performed and a catheter was placed across the aortic valve. Fluorescently labeled color microspheres were injected into the left ventricle at baseline and following a five-minute infusion of dobutamine (10 μg/kg/min) to determine regional differences in myocardial blood flow. A reference blood sample was obtained from a catheter in the femoral artery during administration of the microspheres. A sternotomy was then performed, the heart was excised, and tissue samples were obtained in the ischemic anterior wall and remote regions, for blood flow, proteomic and histologic analysis.

### Isolation of nuclear and mitochondrial samples from cardiac tissue

Cardiac tissue was homogenized using the gentleMACS homogenizer (Miltenyi Biotec; Bergisch Gladbach, Germany), and mitochondrial fractions were tagged with magnetic beads conjugated to TOM22, allowing for magnetic separation and isolation of mitochondria. Once isolated, mitochondria were suspended in RIPA buffer with 1x protease inhibitor for downstream analysis. Nuclear fractions were isolated using commercially available kits (ThermoFisher, Waltham, MA) as described in the manufacturer’s provided protocol. Both mitochondrial and nuclear protein concentrations were determined using a standard BCA assay.

### Western blot

Nuclear and mitochondrial fractions were run in denatured and reduced conditions using equal amounts of protein on a 10% tris-glycine gel and transferred to a nitrocellulose membrane using the semi-dry TransBlot Turbo system (Biorad; Hercules, CA). Membranes with nuclear isolates were probed with a primary PGC1α antibody (ab54481, Abcam; Cambridge, UK), and membranes with mitochondrial isolates were probed with primary OXPHOS antibody (ab110413, Abcam) and incubated with a near-infra-red secondary antibodies (LI-COR Biosciences; Lincoln, NE) for detection with the LiCor Odyssey Imager. Band density was quantified using Image Studio software (LI-COR) and normalized to total protein (REVERT total protein stain, LI-COR).

### Proteomic analysis with TMT™

Identification and relative quantification of mitochondrial proteins isolated from swine are performed using TMT™ (tandem mass tag) reagents (ThermoFisher) in conjunction with liquid chromatography and tandem mass spectrometry (LC-MS/MS). The TMT™ isobaric reagent labels all primary amines to yield labeled peptides that are identical in mass and are also identical in single MS mode. We compared protein concentrations from mitochondria of the LAD region from HM hearts (either CoQ10 or placebo) to a normal control in two 10-plex studies using the same normal control in both studies, as we have previously described [25]. To label mitochondrial proteins from individual samples, isolates are centrifuged and 40 μg of protein from each sample are rehydrated in 0.5 M triethylammonium bicarbonate buffer, pH 8.5, denatured, reduced, alkylated, trypsin digested independently in parallel and labeled with TMT™ reagents. After labeling the peptides, all samples are pooled and dried in vacuo prior to liquid chromatography and tandem MS. To reduce the complexity of the tryptic peptides, peptides are separated by a strong cation exchange into 16 fractions. The proteins from each fraction are separated by reversed phase high performance liquid chromatography (HPLC) and then introduced (on-line) into a mass spectrometer. The capillary HPLC system is interfaced with an Orbitrap Fusion mass spectrometer (ThermoFisher) via a nano-electrospray ionization source. Protein identification and relative quantification are carried out using Proteome Discoverer 2.1 (ThermoFisher) and Scaffold 4.7.3 (Proteome Software, Portland, OR) software programs. MS/MS data are searched against a reference protein species-specific sequence database: UniProt (uniprot.org) plus the common contaminants protein sequences. False discovery rates for protein, peptide and spectral matches are estimated in Scaffold. The average protein relative quantification is calculated from the TMT™ reporter ions for each reagent pair. This relative quantification is based on the ratio of the mitochondrial protein abundance from the LAD region in each CoQ10 treated animal compared to the mitochondrial protein abundance from the LAD region of a placebo treated animal. This ratio indicates whether protein relative abundance is increased or decreased.

### Statistics

All data are presented as mean ± SEM. Differences between the means of multiple groups were compared using two-way ANOVA with Tukey’s test for multiple comparisons, and comparisons between two groups were tested using student’s T-test using GraphPad Prism (La Jolla, CA). A *p*-value < 0.05 was considered significant unless otherwise stated. The method for reporting statistical differences in protein abundances between sample categories of TMT data is permutation testing, to which multiple hypothesis testing corrections can be applied. Statistical analysis of TMT data was done using Scaffold software (Proteome Software, Inc), and significance was determined using the Benjamini-Hochberg test.

## Results

### Swine model of chronic cardiac ischemia represents clinical HM

**Fig. 2:**
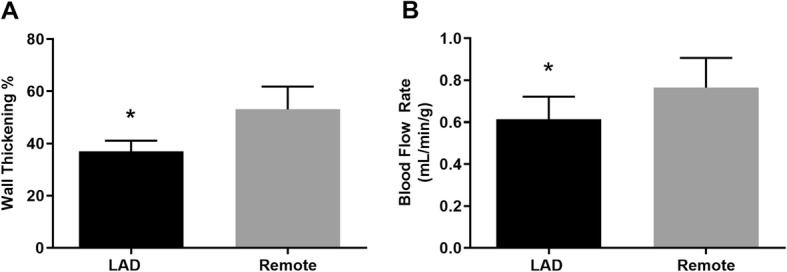
Characterization of the animal model of HM. **a** ECHO measurements of systolic wall thickening show reduced function in the region supplied by the constricted LAD artery (*p* = 0.05); **b** measurements of regional blood flow indicate reduced flow in the region supplied by the constricted LAD artery (*p* = 0.02). Values shown are mean ± SEM

Twelve weeks following instrumentation with a constrictor on the LAD artery, 2D ECHO images demonstrated a decrease in regional wall thickening in the distribution of the LAD artery as compared to a control remote region (*p* <  0.05) (Fig. 2a). Baseline regional blood flow in the same region as measured by fluorescent microspheres is also reduced as measured at the terminal study (16 weeks) in untreated animals with HM (Fig. 2b). Histological analysis of HM tissue with trichrome staining did not show evidence of necrosis (data not shown). These observations represent the clinical definition of HM: reduced flow and reduced function with preserved viability.

### CoQ_10_ administration and regional wall thickening in the chronically ischemic LAD region

**Fig. 3:**
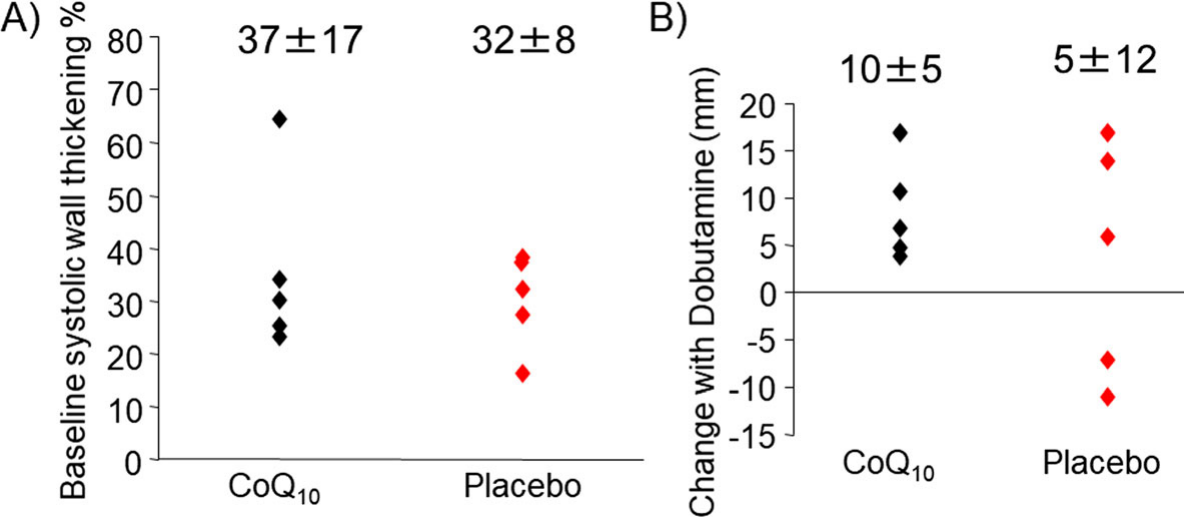
Regional function following 30 days CoQ10 supplementation in HM pigs. ECHO measurements of systolic wall thickening **a** at rest indicate that regional impairment persists with CoQ10 administration, though one animal showed functional improvement. **b** Under increased work with dobutamine infusion, CoQ_10_ treated animals showed increased ability to respond to the increase in demand

Following four weeks of treatment with dietary CoQ10, ECHO was repeated to measure regional function at baseline and during dobutamine infusion. Baseline systolic wall thickening was slightly improved in CoQ10 treated animals, though this improvement did not reach statistical significance (Fig. 3a). Of interest, all CoQ10 treated animals showed an increase in systolic wall thickening during dobutamine infusion (10 μg/kg/min), as compared to the placebo group in which 40% of the animals were unable to respond to the increase in work (Fig. 3b).

### CoQ10 increases expression of metabolic peptides

**Fig. 4:**
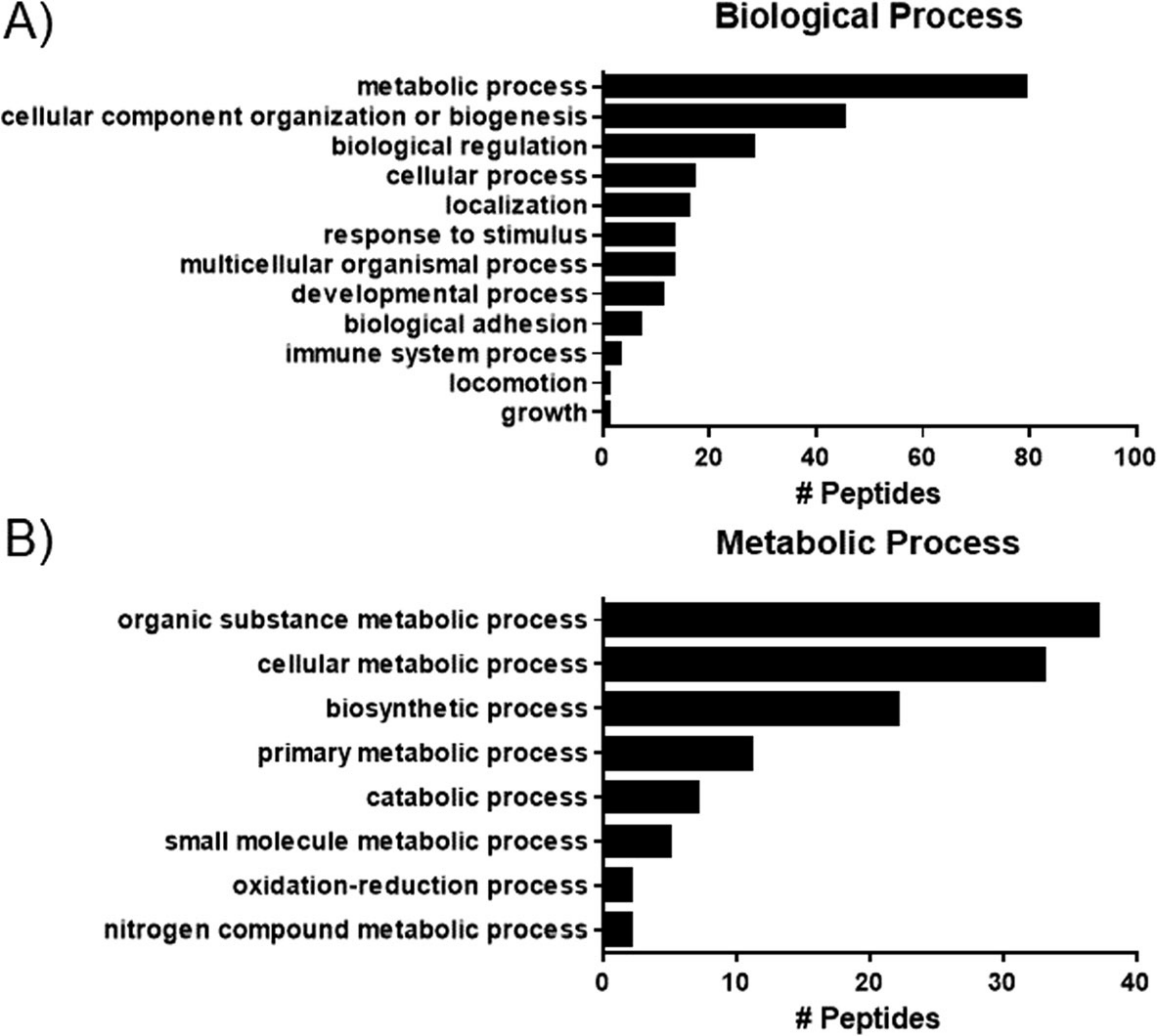
Pathway analysis (PANTHER database) of proteins significantly increased with CoQ10 supplementation as measured by TMT proteomics. **a** pathway analysis of biological processes indicated that upregulated peptides were predominantly involved in metabolic and cellular component biogenesis pathways. **b** Analysis of sub-pathways of metabolic processes indicate that these peptides were predominantly involved in organic substance and cellular metabolic processes

**Fig. 5:**
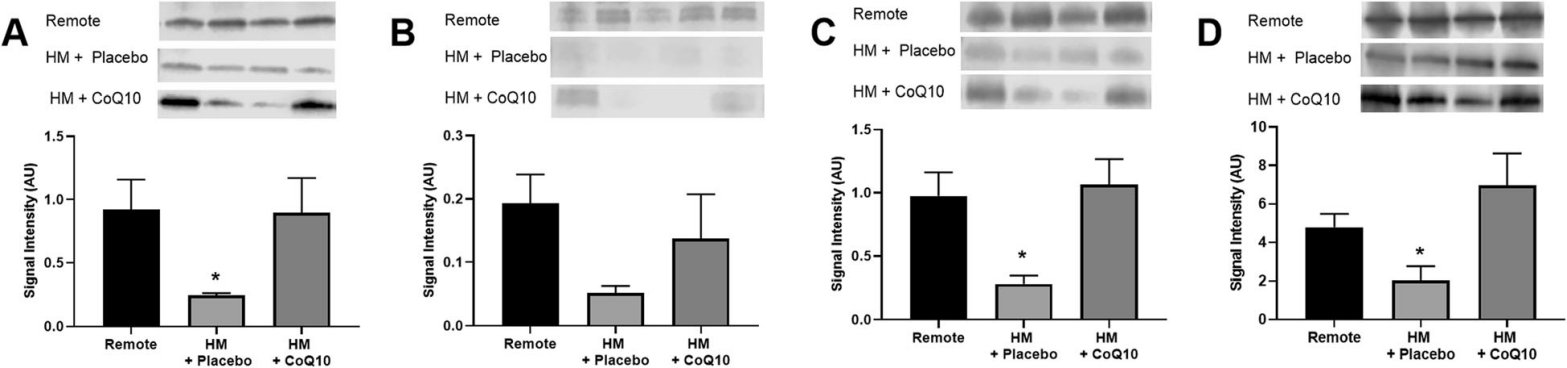
Dietary CoQ10 administration in HM pigs increased expression of ETC proteins as compared to HM pigs treated with placebo. ETC complexes were measured from mitochondrial fractions isolated from the LAD region of HM hearts and probed by western blot. Representative blots are included for each protein measured. Western blots show a significant increase in the protein levels of **a** NADH Dehydrogenase, **c** Cytochrome C Oxidase, and **d** ATP synthase. Succinate Dehydrogenase **b** was increased with CoQ10, but the change was not considered significant. (*P* <  0.05). Values shown are mean ± SEM

Using TMT proteomics, 1823 peptides from mitochondrial isolates of hibernating cardiac tissue were identified. Following identification, peptides were quantified and normalized to control mitochondria from healthy pig hearts. Using Scaffold and the Benjamini-Hochberg test we found 75 peptides were significantly increased following CoQ10 supplementation. Proteomic pathway analysis with PANTHER [26, 27] indicated that increased proteins were predominantly part of the metabolic process (Fig. 4a), and within that process, that peptides were predominantly part of the cellular metabolic process and organic substance metabolic process (Fig. 4b). Peptides of interest relating to the electron transport chain and ATP production that were significantly increased are listed in Table 1, and confirm the findings from Fig. 5.

**Table 1:** List of metabolic peptides significantly upregulated following CoQ10 supplementation as measured by TMT proteomic analysis

Peptide	Accession Number	Permutation Test (*p*-value) Benjamini-Hochberg (*p* < 0.00834)	Log Fold Change (HM + CoQ10 / HM + Placebo)
ATP binding cassette subfamily B member 8	I3LMV8_PIG	0.0037	0.2
ATP synthase subunit alpha	A0A287AGU2_PIG (+ 1)	0.001	0.1
Citrate synthase	CISY_PIG	0.003	0.2
Coenzyme Q10A	F1SLZ5_PIG	0.1	0.2
Coenzyme Q10B	F1SMZ8_PIG	0.034	0.34
Coenzyme Q8A	A0A286ZK64_PIG	0.001	0.28
Coenzyme Q9	I3LHS7_PIG	0.005	0.26
Glutamate dehydrogenase 1	F1SEN2_PIG	0.003	0.19
Malate dehydrogenase	I3LP41_PIG (+ 1)	< 0.0001	0.22
Methylcrotonoyl-CoA carboxylase 2	A0A287B773_PIG	0.003	0.23
Mitochondrial import inner membrane translocase subunit TIM44	A0A287ADX5_PIG	0.00095	0.2
NAD-dependent protein deacylase sirtuin-5	A0A287AF07_PIG	0.001	0.3
NADH:ubiquinone oxidoreductase subunit V3	I3LRR4_PIG [2]	< 0.0001	0.35
NADH-cytochrome b5 reductase	F1S4N2_PIG	0.001	0.23
Oxoglutarate dehydrogenase	F1SSH8_PIG [3]	0.003	0.15
Propionyl-CoA carboxylase alpha	A0A287ALU0_PIG (+ 2)	0.006	0.14
Pyruvate dehydrogenase E1 component subunit alpha	I3LCI2_PIG	0.00019	0.21
Succinate--CoA ligase [ADP-forming] subunit beta	A0A287AR48_PIG (+ 1)	0.001	0.26
Succinate--CoA ligase [GDP-forming] subunit beta	A0A286ZWJ9_PIG	0.00026	0.2
Succinyl-CoA:3-ketoacid coenzyme A transferase 1	SCOT1_PIG	0.003	0.13
Superoxide dismutase	A0A287A4Z2_PIG (+ 1)	0.001	0.2

### CoQ_10_ increases expression of key electron transport chain proteins in the LAD region

Following 30 days of dietary CoQ_10_ supplementation, expression of ETC complex proteins increased as measured by western blot intensity. Specifically, Complex I, NADH Dehydrogenase was decreased in the chronically ischemic LAD region in swine given placebo as compared to a remote, non-ischemic region of the LV. CoQ_10_ administration increased protein expression of Complex I to near normal levels (Fig. 5a; *p* = 0.03). Complex IV, Cytochrome C Oxidase, was similarly decreased in the LAD region as compared to the non-ischemic remote region. CoQ_10_ administration increased protein levels of Complex IV to near normal levels (Fig. 5c; *p* = 0.04). Complex V, ATP Synthase, was also decreased in the LAD region as compared to the non-ischemic remote region. CoQ_10_ administration increased protein expression of Complex V to levels higher than control and significantly different from placebo (Fig. 5d; *p* = 0.028). Complex II, Succinate Dehydrogenase, was lower in the animals given placebo as compared to animals given CoQ_10_ treatment, but these differences did not reach statistical significance (Fig. 5b).

### CoQ_10_ increases expression of nuclear-bound PGC1α in the LAD region

**Fig. 6:**
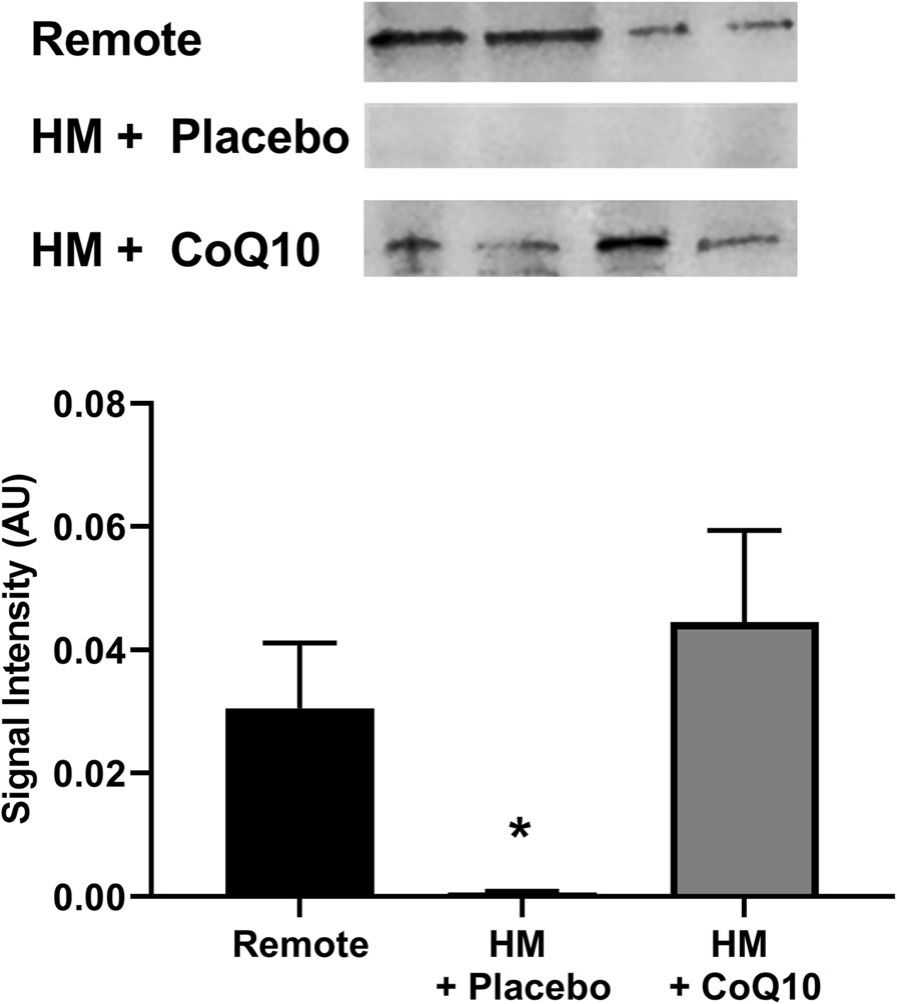
Dietary CoQ10 in HM pigs significantly increased expression of nuclear-bound PGC1α as measured by western blot. Active levels of PGC1α are significantly decreased in HM tissue as compared to the non-ischemic remote region. CoQ10 administration significantly increased expression of PGC1α in the HM region (*p* <  0.05). Representative blot images for each group are pictured. Values shown are mean ± SEM

Using isolated nuclear fractions of cardiac tissue, active, nuclear-bound PGC1α levels were significantly decreased in the LAD region as compared to the non-ischemic remote region. Administration of CoQ_10_ significantly increased levels of active PGC1α in the LAD region (Fig. 6, *p* = 0.01).

### CoQ_10_ increases mitochondrial expression of antioxidant proteins in the LAD region

Using Scaffold to analyze TMT data from mitochondrial fractions, we found that several key antioxidant proteins had increased expression in response to dietary CoQ_10_ supplementation (Table 2). Increases in superoxide dismutase and aldehyde dehydrogenase were significantly increased (*p* <  0.00834) as determined by the permutation test with Benjamini-Hochberg test. Additional antioxidants, including glutathione peroxidase, thioredoxin reductase, and glutathione-disulfide reductase were also increased, but the changes did not reach statistical significance.

**Table 2:** List of antioxidant peptides significantly upregulated following CoQ10 supplementation as measured by TMT proteomic analysis

Peptide	Accession Number	Permutation Test (*p*-value) Benjamini-Hochberg (*p* < 0.00834)	Log Fold Change (HM + CoQ10/HM + Placebo)
Glutathione peroxidase	A0A287AG70_PIG	0.029	0.48
Superoxide dismutase	A0A287A4Z2_PIG (+ 1)	0.001	0.2
Aldehyde dehydrogenase 6	F1S3H1_PIG	0.002	0.17
Superoxide dismutase [Cu-Zn]	SODC_PIG	0.87	0.03
Glutathione S-transferase kappa	F1SRV4_PIG	0.6	0.03
Cluster of Aldehyde dehydrogenase	F1SDC7_PIG [4]	0.54	0.03
Alcohol dehydrogenase, iron containing 1	F1RTZ1_PIG	0.62	0.02
Thioredoxin reductase 2	A0A287BQ74_PIG (+ 1)	0.99	0.01
Glutathione-disulfide reductase	F1RX66_PIG	0.93	0.01

### CoQ10 supplementation did not significantly alter lipid peroxidation

Measurement of MDA in whole tissue homogenates showed an increase in lipid peroxidation in HM animals as compared to non-ischemic tissue (2.479 ± 0.5 vs. 3.6 ± 1 μM). Administration of CoQ10 for 30 days lowered MDA content (3.6 ± 1 vs. 3.2 ± 1.1 μM), though this decrease did not reach statistical significance.

## Discussion

The principal finding of this study is that daily supplementation with CoQ_10_ in chronically ischemic HM increases the mitochondrial expression of anti-oxidant peptides, as well as key proteins within the ETC potentially via enhanced expression of active nuclear-bound PGC1α. The novelty of this study lies in being the first non-infarct, non-reperfusion model of heart disease in a swine model to demonstrate the potential utility of dietary CoQ10 supplementation by improving proteomic measurements of mitochondrial health and antioxidant status. These alterations influence the mitochondrial dysfunction that is characteristic of HM and not addressed by the standard therapy of revascularization. Although we were unable to show a statistically different change in regional wall thickening either at baseline or during inotropic stimulation, recruitment in thickening was observed in all pigs receiving CoQ10 and only 60% of the pigs that received placebo. It is conceivable that the observed enhancement in expression of ETC and antioxidant proteins have minimized oxidant stress while facilitating ETC protein activity for ATP production.

**Fig. 7:**
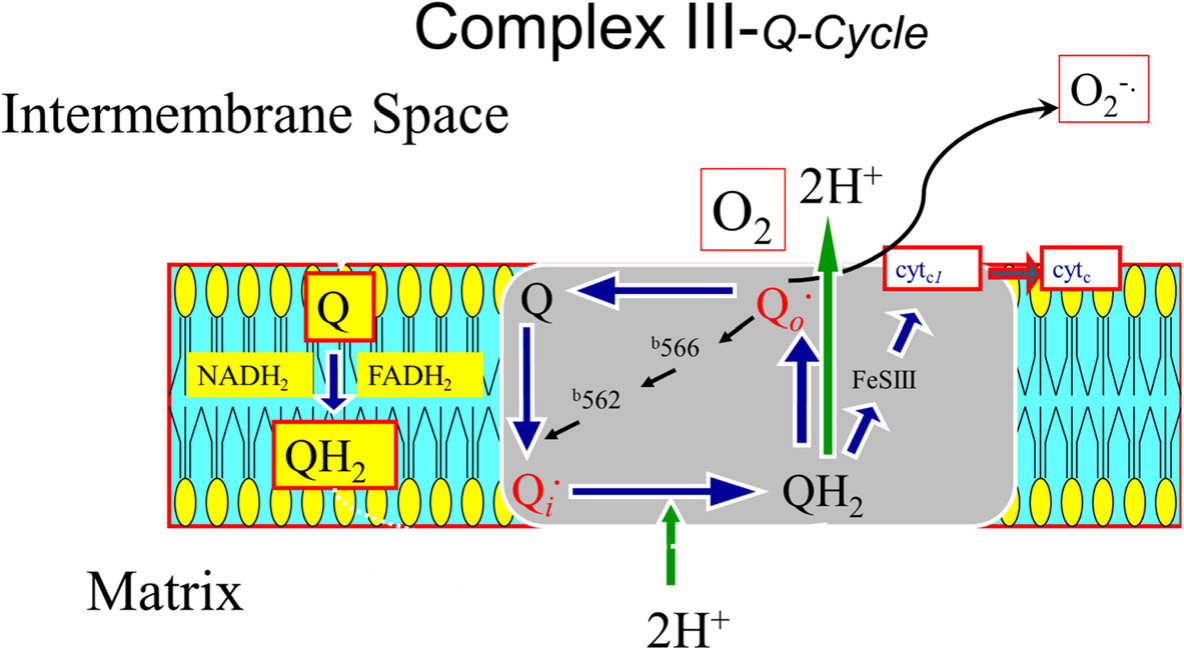
Proposed mechanism of the antioxidant properties of CoQ10. A schema demonstrates a potential mechanism for the addition of CoQ_10_ in the diet for 4 weeks and how enhanced electron transport chain function through Complex III (Q-cycle), reduces the generation of reactive oxygen species into the intermembrane space

In our swine model of HM, we have established the characteristic alterations in the mitochondrial proteome and function, including decreased expression of ETC proteins and impaired respiration that persist despite revascularization [11, 25, 28, 29]. In this study, we aimed to address these mitochondrial abnormalities with the treatment of dietary CoQ_10_ in HM pigs. CoQ_10_ is a lipid-soluble benzoquinone with 10 isoprenyl units in its side chain and plays a key role in the transport of electrons and the synthesis of ATP within the ETC of myocytes (Fig. 7), making it an ideal target for enhancement of impaired mitochondrial respiration and ATP production.

Following CoQ10 treatment, we observed an increase in the expression of ETC proteins within HM regions in which they had previously been depressed. This enhancement would be expected to facilitate ETC function and ATP production, improving bioenergetics while reducing oxidant stress. PGC1α was also enhanced following CoQ10 treatment, and its signaling is known to increase key proteins related to mitochondrial fusion, which is responsible for the process of mitochondrial biogenesis [16, 20, 30–32]. PGC1α is an important regulator of mitochondrial biogenesis and protein expression and is persistently reduced in our model of HM despite CABG [15]. During the aging process, PGC1α is decreased and is associated with reduced levels of glutathione (GSH) and increased levels of oxidant stress within muscle tissue [21]. In studies of isolated C2C12 skeletal muscle cells, supplementation of CoQ_10_ with α-lipoic acid enhanced PGC1α expression while increasing genes encoding proteins involved in glutathione synthesis, recycling, and metabolism [33]. These findings are consistent with those from a rat model of pharmacologically induced seizures, where administration of CoQ_10_ increased expression of PGC1α levels 3-fold and reduced oxidant stress markers [22]. It has been suggested that CoQ_10_ increases the expression and activity of PGC1α by its activation of the cAMP response element binding protein and adenosine monophosphate-activated protein kinase (AMPK) phosphorylation [34].

### Cellular protection of CoQ_10_ against oxidant stress

Experimental work from various animal models and clinical studies support the notion that supplementation with CoQ_10_ has value in reducing oxidant stress. In a rat model of Alzheimer’s disease, cultured cortical neuron induced-damage by exposure to amyloid-beta can be inhibited with the addition of CoQ_10_, and reduce reactive oxygen species, through a mechanism involving activation of the PI3-K/Akt survival pathway [35]. In a swine model, dietary supplementation of CoQ_10_ (5 mg/kg/day) for 30 days increased the myocardial content of ubiquinone in isolated mitochondria by 30%. When the pig hearts were placed on cardiopulmonary bypass and subjected to 30 min of regional ischemia-reperfusion, CoQ_10_ treated hearts showed improved LV function, lower levels of creatine kinase release and reduced levels of MDA content, a marker of oxidant stress within the post-ischemic tissue [24]. Taken together, these data support the concept that CoQ_10_ provides a key role as an antioxidant in cardiomyocytes by enhancing ETC exchange within mitochondria as well as increasing expression of antioxidant proteins, thus reducing the accumulation of oxidant stress within cardiac tissue. This is supported by our own findings that antioxidant peptides are enhanced in the mitochondria of HM hearts following CoQ10 treatment. Despite this upregulation of antioxidant peptides, we did not observe a corresponding decrease in lipid peroxidation following CoQ10 treatment. This may be explained in part by the time points that were assessed. Repeated measurements following longer periods of treatment may result in reduced lipid peroxidation, and an increased dose of CoQ10 may enhance the anti-oxidant effect.

### Clinical studies of CoQ_10_ and improved outcomes

Although we did not see a significant change in regional wall thickening, some functional improvements were observed. It is conceivable that repetitive-supply demand mismatch and excess oxidant stress might have been further mitigated with a longer period of dietary administration or increased dose of CoQ_10_. This may be particularly important to address sudden death in this animal model, which we and others have observed can occur after the three months following instrumentation [3, 36] if no treatment is administered. Among patients undergoing cardiac surgery, CoQ_10_ supplementation reduced the need for inotropic drugs following the operation with a lower incidence of arrhythmias noted [37]. Among aging Swedish people, CoQ_10_ (200 mg/day) with selenium (200 μg as selenized yeast) reduced cardiovascular mortality at 4-years, as well as 10-years following randomization [38]. In addition to these observations, the results of the Q-SYMBIO trial, showed that among patients with stable congestive heart failure, there was a long-term benefit of chronic administration of CoQ_10_ (300 mg/day) versus placebo. The trial was a double-blind, randomized controlled trial and demonstrated a significant long-term reduction in major cardiovascular end-points with treatment [39].

Recently, our group has reported that CoQ10 administration for three days prior to vascular surgery lowers perioperative NT-Pro BNP, which correlates with reduced myocardial injury [40]. Data from this study taken together with the results of our clinical work suggests that while there may not be a detectable direct improvement in regional cardiac function following CoQ10 supplementation, there is benefit to heart patients. Our proteomic results along with the biomarker changes found in the clinical study show that the effect of CoQ10 may be in providing an anti-oxidant effect and improving mitochondrial health rather than having a direct effect on cardiac function and contractility.

### Limitations

This model of HM requires the use of juvenile swine as they have LAD vessels that will gradually increase in size as they grow, creating the gradual stenosis needed to initiate the HM phenotype. An additional limitation of our model is the use of all female animals, which were chosen to reduce variability in a small study as well as to avoid safety issues of social housing with large, tusked males. Even with the use of all female age-matched swine, there remains some biological variability between subjects which is noticeable in a small study such as this.

This model utilizes a single-vessel stenosis which provides a reproducible injury with low mortality rate. However, it is rare to see a clinical case of heart disease with only a single vessel affected.

### Summary

We have shown that administration of CoQ10 in the diet enhances the expression of key ETC proteins, nuclear-bound PGC1α, and important anti-oxidant proteins within the mitochondria. We showed a tendency towards enhanced contractile reserve during inotropic stimulation, though the differences did not reach statistical significance. A plausible mechanism for the observed enhancement in expression of mitochondrial ETC and antioxidant proteins is through enhanced mitochondrial biogenesis via PGC1α signaling, which has previously been demonstrated with the administration of CoQ10 [33]. Future studies should include longer treatment times and increased doses of CoQ10 to determine the effect on functional recovery in HM. These results suggest a potential therapeutic role for CoQ10 in promoting anti-oxidant status and improving mitochondrial function in HM.

## Data Availability

The datasets used and/or analyzed during the current study are available from the corresponding author on reasonable request.

## Abbreviations

AMPK: Adenosine monophosphate-activated protein kinase
ANOVA: Analysis of variance
ATP: Adenosine triphosphate
BCA: Bicinchoninic acid assay
CABG: Coronary artery bypass graft
CAD: Coronary artery disease
CoQ10: Coenzyme q10
ECHO: Echocardiography
ETC: Electron transport chain
GSH: Glutathione
HM: Hibernating myocardium
HPLC: High performance liquid chromatography
LAD: Left anterior descending artery
LC-MS/MS: Liquid chromatography and tandem mass spectrometry
MDA: Malonaldehyde
PGC1α: Peroxisome proliferator activated receptor gamma coactivator 1 alpha
ROS: Reactive oxygen species
TMT: Tandem mass tag
